# Physical activity for people living with dementia: carer outcomes and side effects from the perspectives of professionals and family carers

**DOI:** 10.1007/s40520-020-01636-7

**Published:** 2020-07-03

**Authors:** Ana-Carolina Gonçalves, Sara Demain, Dinesh Samuel, Alda Marques

**Affiliations:** 1 School of Health Sciences, Faculty of Environmental and Life Sciences, University of Southampton, Southampton, UK; 2 Western Sussex Hospitals NHS Foundation Trust, Worthing, UK; 3 School of Health Professions, Peninsula Allied Health Centre, University of Plymouth, Plymouth, UK; 4 Respiratory Research and Rehabilitation Laboratory (Lab3R), School of Health Sciences (ESSUA) and Institute for Research in Biomedicine (iBiMED), University of Aveiro, Agras do Crasto, Campus Universitário de Santiago, Building 30, 3810-193 Aveiro, Portugal

**Keywords:** Physical activity, Dementia, Caregivers, Adherence, Side effects

## Abstract

**Background:**

Adherence to physical activity is challenging for people living with dementia, and largely dependent on carers’ involvement. Carers are likely to support physical activity based on their perceived balance between benefits and potential side effects of such intervention for both patients and themselves. Professionals also have a role in terms of optimising such interventions not only for people with dementia but also their carers.

**Aims:**

The present study aimed to identify the priorities of carers and professionals regarding (1) outcomes of physical activity for people living with dementia on carers and (2) side effects on patients and carers.

**Methods:**

This was a two-round prioritisation exercise. In round one, participants were asked to rank, from most to least important, 2 lists of outcomes generated in a previous systematic review and qualitative study: (i) 10 outcomes on carers; (ii) 17 side effects on patients and carers. In round two, participants were asked to consider their own ranking in round one against the overall group ranking and re-rank both lists.

**Results:**

36 carers and 39 professionals completed both rounds. The carer outcomes ranked as highest priority were “carer feeling positive and satisfied”, “carer improving wellbeing” and “making lives of carers easier”. The most undesirable side effects were “becoming agitated and confused”, “falling over” and “feeling discomfort and pain”.

**Discussion and conclusions:**

Carers and professionals value the potential reduction in carer burden that may occur as a consequence of the person with dementia engaging in physical activity. Behavioural and psychological symptoms, falls and pain are the most undesirable side effects of physical activity. Future research should aim to address, and consistently report on these outcomes.

**Electronic supplementary material:**

The online version of this article (10.1007/s40520-020-01636-7) contains supplementary material, which is available to authorized users.

## Introduction

By 2050, dementia is predicted to affect 131.5 million people worldwide [1]. In the absence of a cure, there is a need for interventions aimed at improving the care of those living with the condition [2]. Physical activity is one such intervention, which has received increased research attention in the last decade [3], due to its promising benefits, including potential improvements in independence in activities of daily living [4, 5], balance [6], physical performance [5] and carer burden [6]. However, due to impairments in cognition and possible loss of motivation [7], professionals may find it challenging to promote and maintain adherence to physical activity in this patient group. People living with dementia are known to be more sedentary than their cognitively healthy peers [8], and largely depend on their carers to engage in physical activity [7]. Informal carers are, therefore, key stakeholders in physical activity interventions for people living with dementia. However, carers of people living with dementia experience higher carer burden than carers of people with other health conditions [9] and expecting them to organise and promote physical activity for their loved one may be unrealistic [7]. Previous research suggests that when designing physical activity interventions, professionals need to focus on carer satisfaction if they wish to optimise adherence [10]. It has also been noticed that carers’ attitudes, feelings and perceptions about physical activity are associated with patients’ levels of physical activity [11]. Therefore, from the perspective of carers, the perceived benefit and subsequent adherence to physical activity are likely to be a trade-off between the potential benefits to patients and carers themselves and the possibility of negative side effects. Professionals delivering physical activity are also key stakeholders in this process, as they may be able to identify the positive outcomes of interventions, as well as side effects on patients and carers and, therefore, adapt (or even decide to discontinue) physical activity interventions according to such outcomes [12].

A core set of positive outcomes of physical activity for people living with dementia has recently been established [13], including outcomes such as “preventing falls”, “enjoying the moment” and “staying healthy and fit”. This core outcome set aims to increase consistency in the reporting of positive outcomes of physical activity for people living with dementia and thus fast-track intervention guidelines. However, the most important outcomes of physical activity interventions for people living with dementia on carers (e.g. carer improving wellbeing or making friends and getting support through the participation of the person living with dementia in physical activity) and the most undesired negative side effects of physical activity (e.g. becoming agitated or experiencing a fall while being active) have not yet been identified. This leaves negative side effects and carer outcomes at risk of being overlooked or inconsistently reported, limiting the inclusion of these important outcomes in literature reviews, meta-analyses and guidance to practice. The present study is a prioritisation exercise, aiming to supplement the already established core outcome set, by defining the priorities of carers and professionals regarding (1) possible positive outcomes that physical activity for people living with dementia may have on carers and (2) negative side effects of physical activity on people living with dementia and/or their carers.

## Materials and methods

### Study design

This prioritisation exercise was nested in a Delphi survey, which aimed to determine a core set of positive patient outcomes to be measured in physical activity interventions for people living with dementia, across settings and stages of disease progression, and which findings have been published previously [13]. In the main Delphi survey, this same group of carers and professionals were joined by people living with dementia to “vote” on the most important positive outcomes of physical activity for people living with dementia. The main Delphi survey was completed via online and paper surveys for carers and professionals, while people living with dementia completed the same Delphi using an innovative face to face card sorting strategy. A more detailed methodological description of this Delphi survey is available elsewhere [13, 14]. It was not considered possible to include negative side effects and carer outcomes in the Delphi survey as this would increase the length of the survey and limit participation of people living with dementia. Therefore, alongside the Delphi consensus process, informal carers and professionals were presented with two lists of outcomes generated from a systematic literature review [3] and a qualitative study [12]: a list of possible negative side effects of physical activity for people living with dementia and their carers; and a list of possible positive carer outcomes that may arise from the person living with dementia taking part in physical activity. Carers and professionals were asked to rank both lists (from most to least important) in a 2-round iterative survey, which is described under “Survey design and data collection”.

### Ethics approval

This study received ethical approval from the Ethics Committee of the Faculty of Health Sciences of the University of Southampton (Ethics number: 19542). Informed consent was ascertained by the completion and return of the surveys.

### Participants and recruitment

Two stakeholder groups were recruited: informal carers of people living with dementia (relatives or friends), referred to as “carers”; and professionals involved in the design, delivery and support of physical activity interventions for people living with dementia, in research and/or clinical practice. Carers were recruited from any location in the United Kingdom and they self-declared: their role as informal carers; the stage of disease progression of the person living with dementia who they cared for; and, the setting where physical activity took place. Professionals who were able to understand written English were recruited from any country in the world and also self-declared their role and experience in dementia care. Recruitment sought volunteers through dementia and carer related charities and support groups (e.g. Alzheimer’s Society; Carers in Southampton), and through professional organisations (e.g. Chartered Society of Physiotherapy). Additionally, a snowball recruitment strategy was adopted [15], where participants were asked to share the survey link or the contact details of the research team with peers who may also be interested in taking part.

The ideal sample size for prioritisation of health outcomes has not been defined. Previous prioritisation studies report sample sizes ranging from 26 [16] to 3000 [17]. In the current study, it was considered feasible to aim for a sample of 40 participants in each stakeholder group to complete the first survey round. The decision for this sample size was made based on previous literature, where a small number (*n* = 23) of homogenous participants in Delphi surveys has proven to be as stable as large computer-generated answers simulating 1000 and 2000 participants [18]. A sample size of 40 in each stakeholder group would, therefore, allow for some heterogeneity in the sample (e.g. carers with different relationships to the person living with dementia; professionals from different backgrounds, etc.) while keeping the study feasible in terms of recruitment, in the time scale available to deliver the study.

### Survey design and data collection

The two lists of outcomes generated from the literature [3] and a previous qualitative study [12] included (i) 10 positive outcomes that supporting physical activity for the person living with dementia may have on carers; (ii) 17 negative side effects of physical activity for the person living with dementia and/or their carers. Patient and public representatives assisted the authors to write these outcomes in lay terms.

The survey was made available in paper (using pre-paid envelops for the return of the surveys) and electronic formats (using the SurveyGizmo software). The surveys were very similar in both formats. In the paper format, the participants were asked to use numbers to rank the outcomes (“1” being the most important). In the electronic format, participants were instructed to click and drag the outcomes in order (with the most important on top, equivalent to ranking position “1”). This was a forced ranking exercise and, therefore, two outcomes could not be ranked with the same number, or dragged and dropped in the same position.

#### Round one

The first round of the survey included demographic questions for sample characterisation purposes, as well as the two lists of outcomes described above. The order in which outcomes were presented was randomised, as the order in which survey items are presented is known to influence participants’ choices [19]. There were 15 randomly ordered versions of the paper surveys that were distributed during recruitment, and the electronic version of the survey automatically randomised the order of the outcomes every time a new participant opened the survey link. In round one, participants were also asked to add any missing outcomes to either list. A glossary with the definitions of each of the outcomes in the survey was available to all participants.

#### Round two

Participants who completed round one, were sent the round two survey in the format they had used to complete round one (either electronic or paper). In round two, outcomes were presented in ranked order from round one. Each participant was reminded of their own round one ranking order choices. New outcomes suggested in round one were added to the list and the glossary was updated accordingly. A detailed booklet showing how outcomes had been ranked per stakeholder group was also made available. Participants were asked to consider the results from the previous round and re-rank the outcomes. This allowed participants to make an informed judgement regarding their final ranking, as the second round included new outcomes introduced by participants in round one, and each individual participant could compare their own priorities against those of other carers and professionals. An example of the paper survey, and a detailed results booklet, also including the glossary definition provided to the participants is available as supplementary material to this paper.

### Data analysis

Descriptive statistics were used for demographic data. These include counts and percentages of the following sample covariates: age, gender, experience supporting people living with dementia per stage of disease progression, setting and country in which physical activity took place.

Outcome ranking data were only considered from round two and therefore only data from participants who completed both survey rounds were included. For each outcome, all ranking positions were summed, and the outcome with the lowest overall ranking number was considered the most important.

It was understood that non-ranked outcomes (left blank by participants in paper surveys) would have been considered less important than those ranked with a number (as some participants wrote notes next to non-ranked outcomes expressing that such outcomes were not applicable to them at all, and were therefore not ranked). Scoring non-ranked outcomes as “zero” would have given them more importance (as in the sum of ranking positions, the lowest overall ranking was considered most important). Therefore, a decision was made to score all non-ranked outcomes as “number of outcomes plus one”. This means that all non-ranked outcomes were counted as less important than the lowest ranked outcome by one.

## Results

### Participant characteristics

A total of 75 participants (36 carers and 39 professionals) completed both survey rounds, out of 91 (44 carers and 47 professionals) who had completed round one. Of the participants who completed both rounds, all professionals completed their surveys electronically, whereas 15 carers chose to take part using the paper format. Both stakeholder groups included participants with experience of supporting physical activity across stages of dementia and activity settings (Table 1).

**Table 1:** Characteristics of participants in the round two survey

	Carer stakeholder group (*n* = 36)	Professional stakeholder groups (*n* = 39)
	*n* (%)	*n* (%)
Gender (male)	12 (33.3%)	10 (25.6%)
Age (years)		
18–29	1 (2.8%)	8 (20.5%)
30–39	0 (0%)	9 (23.1%)
40–49	2 (5.6%)	6 (15.4%)
50–59	12 (33.3%)	12 (30.8%)
60–69	9 (25%)	3 (7.7%)
70–79	10 (27.8%)	1 (2.6%)
Supporting people in the following stages of dementia progression		
Mild to moderate	12 (33.3%)	8 (20.5%)
Moderate to severe	13 (36.1%)	7 (17.9%)
Severe	0 (0%)	2 (5.1%)
All stages	11 (30.6)	26 (66.7%)
Not known	2 (5.6%)	0 (0%)
Supporting activity in the following settings		
Community	34 (94.4%)	35 (89.7%)
Sheltered accommodation	3 (8.3%)	8 (20.5%)
Care or nursing home	12 (33.3%)	22 (56.4%)
Hospital	2 (5.6%)	24 (61.5%)
Country		
England	36 (100%)	35 (89.7%)
Wales	0 (0%)	1 (2.6%)
France	0 (0%)	1 (2.6%)
Portugal	0 (0%)	1 (2.6%)
Brazil	0 (0%)	1 (2.6%)
Singapore	0 (0%)	1 (2.6%)
Relationship to the person living with dementia (one carer cared for more than one person living with dementia)		
Spouse/partner	14 (38.9%)	Not applicable
Adult children	17 (47.2%)	Not applicable
Children in law	4 (11.1%)	Not applicable
Grandchildren	1 (2.8%)	Not applicable
Friends	1 (2.8%)	Not applicable
Professional background (some professionals had a dual role, e.g. physiotherapist and researcher):		
Physiotherapists	Not applicable	14 (35.9%)
Researchers	Not applicable	8 (20.5%)
Members of volunteer organisations	Not applicable	7 (17.9%)
Occupational therapists	Not applicable	6 (15.4%)
Rehabilitation assistants	Not applicable	4 (10.3%)
Social workers	Not applicable	1 (2.6%)
Nurses	Not applicable	1 (2.6%)
Health care support workers	Not applicable	1 (2.6%)

### Ranking results

In round one, participants added two new carer outcomes: “Carer feeling less worried” and “carer living longer”. A total of four new negative side effects were also added: “becoming more disabled”, “forgetting the activity”, “carer feeling heartbroken” and “creating a conflict between the carer and the person living with dementia”. Therefore, 12 positive carer outcomes and 21 negative side effects of physical activity were considered in round two. In this last round, the top three carer outcomes identified by both stakeholder groups were: “carer feeling positive and satisfied”; “carer improving wellbeing” and “making the lives of carers easier”. The three most undesirable side effects across all participants were “becoming agitated and confused”, “falling over” and “feeling discomfort and pain”. When considering the rankings by stakeholder groups, carers ranked the same three negative side effects as the overall group but professionals put “falling over” in the ranking position number four, and “having a bad experience” in third place (Table 2). Refer to supplementary material 2 for a full list of all outcomes ranked by stakeholder group.

**Table 2:** Top three carer outcomes and top three side effects of physical activity from the perspective of carers and professionals

	Sum of ranking positions from all participants (*n* = 75)	Final overall ranking position	Definition, as in the glossary made available to participants
Top three carer outcomes in lay terminology			
Carer feeling positive and satisfied	228	1	Carers feeling positive about the person living with dementia being active, improving and having a fulfilling time. Carers feeling proud of the person living with dementia and seeing them doing activities they used to do in the past. Carer having better self-esteem. In the literature this was linked to confidence in their care abilities and carers’ satisfaction with the intervention
Carer improving wellbeing	230	2	Carer wellbeing and quality of life. Carer having fun
Making the lives of carers easier	319	3	Physical activity may reduce the burden of care in the long-term by: maintaining functional independence of the person with dementia and finding the person living with dementia more agreeable to tasks, lightening the workload that need to be done by the carer; carer accessing support from professionals; and carer experiencing less challenging behaviour, including less sundowning from the person living with dementia. In the short-term, carers’ lives can be made easier by giving the carer a break; time and space to themselves or some respite, while the person with dementia is involved in physical activity and needing less input from the carer
Top three side effects of activity			
Becoming agitated and confused	319	1	Becoming challenging, frustrated, rude or overstimulated during physical activity. Refusing to go back into a care setting after an activity in a different environment. In some cases, activities with these effects were considered not appropriate for the person living with dementia and are often interrupted
Falling over	400	2	Sustaining falls or increasing falls risk by being active. Sustaining injuries after a fall (e.g. fractures) and having to attend emergency care because of falls. Being about to fall, but being able to save oneself. Increasing fear of falling and reduced confidence in walking due to fear of a fall
Feeling discomfort and pain	419	3	Includes joint pain, muscle soreness or stiffness after exercising. Complaining of pain or experiencing physical discomfort during physical activity. Not being able to be as active as usual in the day(s) after the activity

## Discussion

Physical activity interventions for people living with dementia require effort, carer involvement and are not free from potential negative side effects, identified by both carers and professionals. The present study successfully identified the top positive carer outcomes of supporting physical activity for the person living with dementia (i.e. “carer feeling positive and satisfied”; “carer improving wellbeing” and “making lives of carers easier”); as well as the most undesired side effects of physical activity (i.e. “becoming agitated and confused”, “feeling discomfort and pain” and “falling over”), from the perspectives of carers and professionals.

The three carer outcomes ranked as most important by the carers and professionals, relate to key aspects of carer burden, which are well described in the literature about dementia caregiving [20]. Reduced burden of care (“making lives of carers easier”) has been linked to carer sense of competence or self-efficacy (“carer feeling positive and satisfied”) and carer quality of life (“carer improving wellbeing”) [20]. Interventions to reduce carer burden do not always consider physical activity for the person living with dementia as a possible solution [21]. Yet this could be a possibility, as a recent systematic literature review found physical activity for the person living with dementia to be effective at reducing carer burden [6]. However, the relationship between physical activity for the person living with dementia and carer burden may be complex. Relying on carers to organise and support physical activity is likely to increase their workload and perceived burden [7, 22]; however, physical activity for the person living with dementia, without carer involvement, may give carers a break, reducing their burden of care [23]. Moreover, the perceived burden of care may be linked to more than just the number of task carers are required to undertake to make the physical activity happen, but also the way a particular physical activity intervention is designed and delivered. Current carer policy notes that carers value being involved and listened to, in the design of interventions for the person living with dementia [24] and professionals are encouraged to establish a proactive and respectful collaboration with carers [7], which may increase their self-efficacy, decrease the perceived burden of care, ultimately promoting adherence to physical activity.

The key role of carers in engaging the person living with dementia in physical activity was also reflected in how the participants in this study ranked possible negative side effects of physical activity. The three most undesirable side effects of physical activity for the person living with dementia can all be related back to carer burden.

First, behavioural and physiological symptoms of dementia (“increased confusion and agitation”) have been associated with carer burden in previous research [20, 25]. These are also commonly reported negative side effects of physical activity [3] and could be a direct barrier to participation in physical activity [26].

Second, pain (“feeling discomfort and pain”) is a known possible cause of behavioural and psychological symptoms of dementia and also a cause of functional dependence for the person living with dementia [27]. Symptoms of pain in a person living with dementia can, therefore, be indirectly linked to carer burden. Although important, pain has not often been reported as a negative effect of physical activity for people living with dementia [3]. It is unclear if this is because pain has actually been linked to inactivity (rather than activity) [28], or if pain has simply been missed as a side effect, since expressing themselves is challenging and poses difficulties to recognise pain levels in people living with dementia [29]. Further research is needed to understand this further.

Lastly, falls also known to have a major impact on carer burden and carer perceived ability to care [30]. People living with dementia are more likely to fall, be injured and admitted to hospital after a fall than people without dementia [31, 32]. In previous research, falls prevention has been agreed to be amongst the most important positive outcome of physical activity for people living with dementia, and recommended to be measured in all future research in this area [13]. The present study shows that it is important to record falls both as a positive outcome (falls prevention) and a potential side effect (increased falls during activity) in future physical activity intervention for people living with dementia.

### Strengths and limitations

To our knowledge, this was the first study to provide insight into the priorities of carers and professionals regarding both carer outcomes from supporting physical activity for the person living with dementia, and the possible negative side effects of physical activity. This prioritisation exercise was embedded in a Delphi survey, which allowed some anonymous interaction between participants, who were able to reflect on their own ranking based on feedback from other participants. This method also allowed participants to take part remotely, nationally and internationally.

However, this study is not without limitations, including most importantly, the lack of involvement of people living with dementia in this exercise. People living with dementia took part in the primary Delphi survey in which this prioritisation exercise was embedded [13] using novel card sorting strategies to enable them to prioritise outcomes. This was however a lengthy process (on average 30 min per session) and they were therefore not asked to complete these prioritisation tasks, with the aim of minimising fatigue. Future work should include such methods to gather the views of people living with dementia, particularly on the negative side effects of physical activity. Additionally, it is unclear if the characteristics of the participants in this prioritisation exercise are representative of the general population. For instance, a recent national survey of carers of people living with dementia across England (*n* = 325) shows a similar percentage of carers who are spouses/partners to those included in the present study (36.3% in the national survey compared to 38% in the present study), but comparatively more male carers took part in the present study, than in the larger national survey (33% versus 19%) [24]. It is possible that different demographics may be linked to different priorities regarding outcomes of physical activity for people living with dementia. Further, the possible impact of cultural and environmental contexts should not be ignored. In the present study, most participants were from the south of England. It is likely that perceived outcomes of participation in physical activity interventions for people living with dementia may vary in other cultural contexts. Thus, replication of this survey (aided by the survey and glossary made available with this paper) in other parts of the country and indeed the world is highly encouraged. Further research is also encouraged on the impact of possible comorbidities of the person living with dementia on the perceived importance of side effects and carer outcomes, as well as on how interventions can be adapted to address these important outcomes, in different settings and across stages of disease progression.

## Conclusion

Physical activity for people living with dementia is valued by carers and professionals, not only for its benefits for patients, but also for its potential to reduce carer burden. Negative effects, such as behavioural and psychological symptoms of dementia, pain and falls are potentially the most undesirable side effects of physical activity and can also influence perceived burden of care. These outcomes should be consistently reported in future research in this field, to allow professionals and carers to make informed decisions on the safety of the intervention, according to outcomes meaningful to them. Designing interventions that take into account these outcomes on carers and possible negative side effects may influence adherence to physical activity.

## Electronic supplementary material

Below is the link to the electronic supplementary material.

Supplementary file1 (DOCX 68 kb)
