# Supplementary material for: Physical activity for people living with dementia: carer outcomes and side effects from the perspectives of professionals and family carers
Source: Aging Clin Exp Res. 2020 Jul 3;33(5):1267–74. doi: 10.1007/s40520-020-01636-7 (PMC8081678; doi:10.1007/s40520-020-01636-7)
Supplement: Supplementary file 1 — Supplementary file1 (DOCX 68 kb) [file 40520_2020_1636_MOESM1_ESM.docx]

**SELECTING THE MOST IMPORTANT EFFECTS THAT PHYSICAL ACTIVITY FOR PEOPLE LIVING WITH DEMENTIA MAY HAVE ON CARERS**

The list below shows possible positive effects that physical activity for people living with dementia may have **on their carers**. **These effects are presented in the order indicated by participants in the last round. The order you put them on last time is shown in brackets after each effect.** Now, please consider in which order you would like to put them today. Please write the **numbers 1 to 12** in the boxes below, 1 being the most important effect and 12 the least important. Remember you can look at the attached glossary for information on these effects. You can also see more detail on how the participants from the last round put these effects in order in the “Round one survey results” document included in your pack.

1. Carer feeling positive and satisfied (last time you answered: 1).
2. Carer improving wellbeing (last time you answered: 11).
3. Carer making friends and getting support (last time you answered: 1).
4. Making the lives of carers easier (last time you answered: 1).
5. Carer improving mood (last time you answered: 2).
6. Carer feeling less depressed (last time you answered: 1).
7. Carer getting better sleep (last time you answered: 10).
8. Carer having better health (last time you answered: 1).
9. Carer being more active (last time you answered: 11).

(10) Carer walking better (last time you answered: blank).

(11) Carer feeling less worried (NEW)

(12) Carer living longer (NEW)

**SELECTING THE MOST IMPORTANT NEGATIVE EFFECTS THAT PHYSICAL ACTIVITY ON PEOPLE LIVING WITH DEMENTIA AND THEIR CARERS**

The list below shows possible **negative effects** that physical activity may have on the person living with dementia and their carers. **These effects are presented in the order indicated by participants in the last round. The order you put them in last time is shown in brackets after each effect.** Now, please consider in which order you would like to put them today. Please write the **numbers 1 to 21** in the boxes below, 1 being the most important effect and 21 the least important. Remember you can look at the attached glossary for information on these effects. You can also see more detail on how the participants from the last round put these effects in order in “Round one survey results” document included in your pack. Please note that the list continues on the next page.

1. Becoming agitated and confused (last time you answered: 1).
2. Falling over (last time you answered: 1).
3. Feeling discomfort and pain (last time you answered: 10).
4. Having a bad experience (last time you answered: 1).
5. Getting hurt or accidently hurting others (last time you answered: 1).
6. Feeling tired or exhausted (last time you answered: 1).
7. Becoming unwell or having to go to hospital (last time you answered: 18).
8. Feeling dizzy, sick or fainting (last time you answered: 1).
9. Becoming uncomfortable because of shortness of breath (last time you answered: 18).

(10) Getting lost (last time you answered: 1).

(11) Making the lives of carers harder (last time you answered: 1).

(12) Having a heart problem while exercising (last time you answered: 10).

(13) Not being able to sleep after an activity (last time you answered: 18).

(14) Increasing risk of death (last time you answered: 18).

(15) Going to a care home or a nursing home (last time you answered: 18).

(16) Accidently eating or drinking something harmful (last time you answered: 15).

(17) Finding cysts (last time you answered: 18).

(18) Becoming more disabled (NEW)

(19) Carer feeling “heartbroken” (NEW)

(20) Creating a conflict between the carer and the person living with dementia (NEW)

(21) Forgetting the activity (NEW)

**THANK YOU!**

Thank you for completing this survey. Your contribution is very important and your help is most appreciated.

**Now please post this survey using the pre-paid envelope included in your package.**

We will be in touch in the next few months, either with the results of the final consensus or, if a consensus had not been achieved, with a third (and last) survey round.

If you have any questions or would like more information, please contact XXXX by:

**Post:** XXXXX

**Email**: [XXXXXX](mailto:a.c.goncalves@soton.ac.uk)

**Phone:** XXXXXX

| **Supplementary material 2a**: List of all carer outcomes considered during the prioritisation exercise round two, with ranking positions per stakeholder group. | | | | |
| --- | --- | --- | --- | --- |
| **All carer outcomes in lay terminology** | **Final overall ranking position (N=75)** | **Ranking position considering the carer stakeholder group only (N=36)** | **Ranking position considering the professionals stakeholder group only (N=39)** | **Definition, as in the glossary made available to participants** |
| Carer feeling positive and satisfied | 1 | 1 | 2 | Carers feeling positive about the person living with dementia being active, improving and having a fulfilling time. Carers feeling proud of the person living with dementia and seeing them doing activities they used to do in the past. Carer having better self-esteem. In the literature this was linked to confidence in their care abilities and carers’ satisfaction with the intervention. |
|  |  |  |  |  |
| Carer improving wellbeing | 2 | 2 | 1 | Carer wellbeing and quality of life. Carer having fun. |
|  |  |  |  |  |
| Making the lives of carers easier | 3 | 3 | 3 | Physical activity may reduce the burden of care in the long-term by: maintaining functional independence of the person with dementia and finding the person living with dementia more agreeable to tasks, lightening the workload that need to be done by the carer; carer accessing support from professionals; and carer experiencing less challenging behaviour, including less sun downing from the person living with dementia. In the short-term, carers’ lives can be made easier by: giving the carer a break; time and space to themselves or some respite, while the person with dementia is involved in activity and needing less input from the carer. |
|  |  |  |  |  |
| Carer making friends and getting support | 4 | 5 | 4 | Family carers meeting other relatives of people with dementia and developing a network of friendship and peer support. Carers meeting other carers and having their carer role recognised. |
|  |  |  |  |  |
| Carer feeling less depressed | 5 | 6 | 5 | Lack of activity and engagement for the person living with dementia was linked to carer depression. |
|  |  |  |  |  |
| Carer improving mood | 6 | 4 | 7 | Carer feeling happy or being in better mood. |
|  |  |  |  |  |
| Carer feeling less worried | 7 | 9 | 6 | Lack of activity and engagement for the person living with dementia was linked to carer depression. |
|  |  |  |  |  |
| Carer having better health | 8 | 8 | 8 | Includes weight management and overall wellbeing and health. In the literature this was measured as the carer’s use of health and social care services and therefore linked to costs. |
|  |  |  |  |  |
| Carer getting better sleep | 9 | 7 | 9 | Improved carer sleep quality (specific sleep parameters not specified). |
|  |  |  |  |  |
| Carer being more active | 10 | 10 | 10 | Joining in with the person with dementia, adding to their habitual levels of activity. |
|  |  |  |  |  |
| Carer walking better | 11 | 11 | 11 | Carer mobility and balance, ability to walk with less joint pain. |
|  |  |  |  |  |
| Carer living longer | 12 | 12 | 12 | Carer living longer as a result of the person living with dementia taking part in activity. |
|  |  |  |  |  |

Table 2a - highlighted in gray the new outcomes, added by the round 1 participants

| **Supplementary material 2b:** List of all side effects of physical activity, considered during the prioritisation exercise round two, with ranking positions per stakeholder group. | | | | |
| --- | --- | --- | --- | --- |
| **All side effects of activity for patients and carers in lay terminology** | **Final overall ranking position (N=75)** | **Ranking position considering the carer stakeholder group only (N=36)** | **Ranking position considering the professionals stakeholder group only (N=39)** | **Definition, as in the glossary made available to participants** |
| Becoming agitated and confused | 1 | 2 | 1 | Becoming challenging, frustrated, rude or overstimulated during the physical activity. Refusing to go back into a care setting after a physical activity in a different environment. In some cases, physical activities with these effects were considered not appropriate for the person living with dementia and are often interrupted. |
|  |  |  |  |  |
| Falling over | 2 | 1 | 4 | Sustaining falls or increasing falls risk by being active. Sustaining injuries after a fall (e.g. fractures) and having to attend emergency care because of falls. Being about to fall, but being able to save oneself. Increasing fear of falling and reduced confidence in walking due to fear of a fall. |
|  |  |  |  |  |
| Feeling discomfort and pain | 3 | 3 | 2 | Includes joint pain, muscle soreness or stiffness after exercising. Complaining of pain or experiencing physical discomfort during activity. Not being able to be as active as usual in the day(s) after the physical activity. |
|  |  |  |  |  |
| Having a bad experience | 4 | 4 | 3 | Triggering negative emotions or feelings (e.g. embarrassment or unhappiness). Having an unsatisfying experience. Physical activity reinforcing illness and loss of roles (e.g. letting the team down) and therefore having a negative impact on perceptions of wellbeing. |
|  |  |  |  |  |
| Feeling tired or exhausted | 5 | 5 | 6 | Feeling tired, drained, exhausted. Described as something that would stop the physical activity and that should be used to monitor the intensity of the physical activity. |
|  |  |  |  |  |
| Getting hurt or accidently hurting others | 6 | 6 | 5 | Includes injuries to muscles and joints. May result from doing the wrong exercise or overdoing it; or from “bumping into” objects or fellow participants. |
|  |  |  |  |  |
| Making lives of carers harder | 7 | 8 | 8 | Carers may face an increase in workload: being/feeling responsible to offer physical activity and sometimes persuade them to join in activities. For carers, physical activity can imply effort, fatigue and negative emotions of guilt, frustration, embarrassment and worry about patient safety. It may increase risks of negative health outcomes for the carer. |
|  |  |  |  |  |
| Becoming unwell or having to go to hospital | 8 | 10 | 9 | Being hospitalised or admitted to an emergency department. Experiencing a deterioration of overall health or becoming too unwell to continue activity. |
|  |  |  |  |  |
| Feeling dizzy, sick or fainting | 9 | 9 | 10 | Feeling dizzy, nauseous or light-headed. Having a syncopal episode (losing conscientiousness for a moment). |
|  |  |  |  |  |
| Becoming uncomfortable because of shortness of breath | 10 | 7 | 11 | Shortness of breath that is uncomfortable or more exacerbated than in normal exercise. Needing to “catch one’s breath” and interrupting the physical activity because of it. |
|  |  |  |  |  |
| Creating a conflict between the carer and the person living with dementia | 11 | 13 | 7 | Triggering an argument during a physical activity. Sometimes the conflict can start from the carer trying to guide the person living with dementia to do a physical activity that he/she refuses to do. Impacting on the relationship between the carer and the person living with dementia, where the person living with dementia is “being told” what to do and has less choice. |
|  |  |  |  |  |
| Getting lost | 12 | 12 | 12 | Not being able to find the way back after a physical activity. Having to use technology to find the person with dementia. |
|  |  |  |  |  |
| Having a heart problem while exercising | 13 | 11 | 13 | Suffering a Transient Ischemic Attack or developing cardiac pathology. This is also a reason for caution when involving a person with dementia in physical activity. |
|  |  |  |  |  |
| Not being able to sleep after an activity | 14 | 14 | 15 | This was linked to possible changes of environment or routine caused by the physical activity (e.g. coming back to care after having been on holiday). |
|  |  |  |  |  |
| Becoming more disabled | 15 | 17 | 16 | Becoming or feeling more disabled while doing physical activity after the diagnosis of dementia. |
|  |  |  |  |  |
| Increasing risk of death | 16 | 16 | 17 | Mortality. Number of deaths during physical activity or within the time period while the person with dementia was a participant in a physical activity intervention. |
|  |  |  |  |  |
| Going to a care home or nursing home | 17 | 15 | 19 | Being placed into an institution for permanent full-time care as a consequence of deteriorating health during the physical activity, or resulting in an interruption of participation in physical activity. |
|  |  |  |  |  |
| Carer feeling "heartbroken" | 18 | 20 | 14 | Carer having a sense of loss while seeing the person living with dementia being physically active. |
|  |  |  |  |  |
| Forgetting the activity | 19 | 18 | 18 | Offering physical activity that the person with dementia is then not able to recall. |
|  |  |  |  |  |
| Accidently eating or drinking something harmful | 20 | 19 | 20 | Eating or drinking something that should not be ingested, by confusing it with real food or drink while being physically active (e.g. mistaking a cleaning product with an energy drink while in the gym; eating something from the garden while outdoors). |
|  |  |  |  |  |
| Finding cysts | 21 | 21 | 21 | Right ganglion cyst discovered in research participant of a physical activity intervention. The link between this and the activity was not made clear but the discovery of the cyst was considered a non-serious adverse event possibly related to study intervention. |
|  |  |  |  |  |

Table 2b - highlighted in gray the new outcomes, added by the round 1 participants
